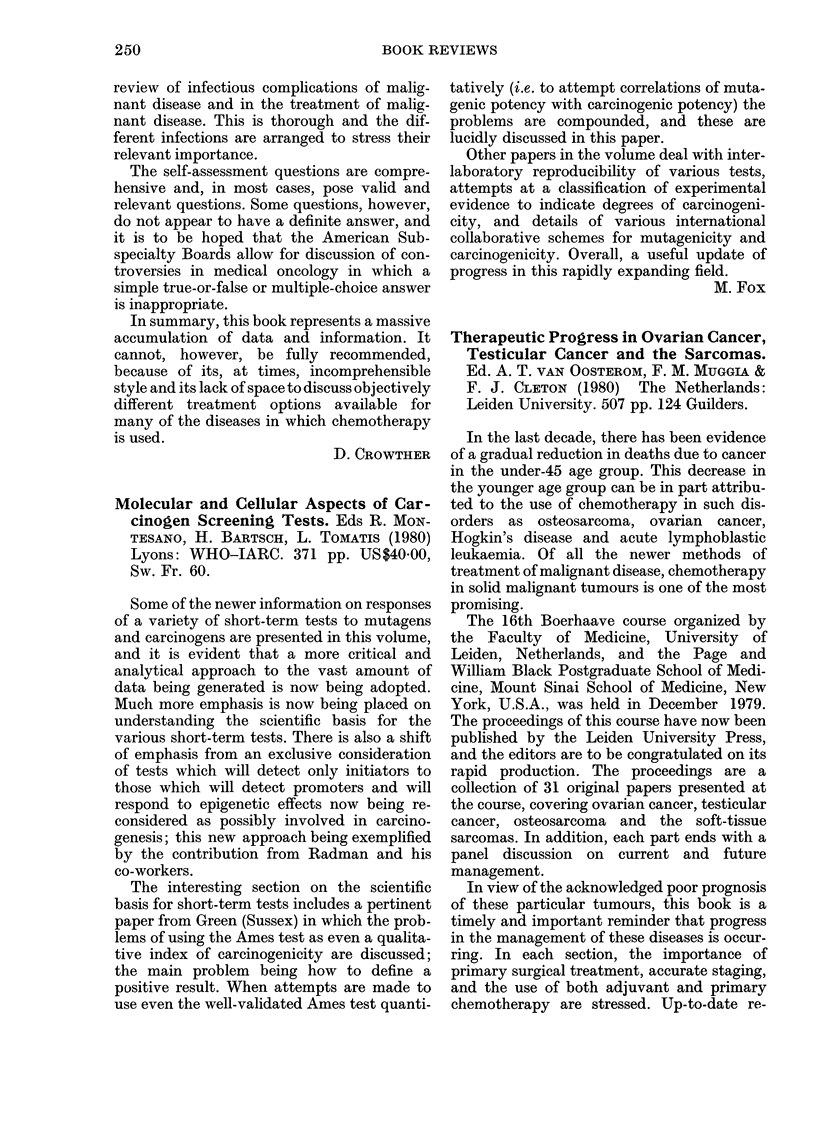# Molecular and Cellular Aspects of Carcinogen Screening Tests

**Published:** 1981-02

**Authors:** M. Fox


**Molecular and Cellular Aspects of Carcinogen Screening Tests.** Eds R. MONTESANO, H. BARTSCH, L. TOMATIS (1980) Lyons: WHO–IARC. 371 pp. US$40·00, Sw. Fr. 60.

Some of the newer information on responses of a variety of short-term tests to mutagens and carcinogens are presented in this volume, and it is evident that a more critical and analytical approach to the vast amount of data being generated is now being adopted. Much more emphasis is now being placed on understanding the scientific basis for the various short-term tests. There is also a shift of emphasis from an exclusive consideration of tests which will detect only initiators to those which will detect promoters and will respond to epigenetic effects now being reconsidered as possibly involved in carcinogenesis; this new approach being exemplified by the contribution from Radman and his co-workers.

The interesting section on the scientific basis for short-term tests includes a pertinent paper from Green (Sussex) in which the problems of using the Ames test as even a qualitative index of carcinogenicity are discussed; the main problem being how to define a positive result. When attempts are made to use even the well-validated Ames test quantitatively (*i.e.* to attempt correlations of mutagenic potency with carcinogenic potency) the problems are compounded, and these are lucidly discussed in this paper.

Other papers in the volume deal with interlaboratory reproducibility of various tests, attempts at a classification of experimental evidence to indicate degrees of carcinogenicity, and details of various international collaborative schemes for mutagenicity and carcinogenicity. Overall, a useful update of progress in this rapidly expanding field.

M. FOX